# A spatial analysis of seagrass habitat and community diversity in the Great Barrier Reef World Heritage Area

**DOI:** 10.1038/s41598-021-01471-4

**Published:** 2021-11-16

**Authors:** Alex B. Carter, Catherine Collier, Emma Lawrence, Michael A. Rasheed, Barbara J. Robson, Rob Coles

**Affiliations:** 1 Centre for Tropical Water & Aquatic Ecosystem Research (TropWATER), James Cook University, Building E1.016A, P.O. Box 6811, Cairns, QLD 4870 Australia; 2 Commonwealth Scientific and Industrial Research Organisation (CSIRO) Data61, Brisbane, Australia; 3 Australian Institute of Marine Science and AIMS@JCU, Townsville, Australia

**Keywords:** Ecology, Environmental sciences

## Abstract

The Great Barrier Reef World Heritage Area (GBRWHA) in north eastern Australia spans 2500 km of coastline and covers an area of ~ 350,000 km^2^. It includes one of the world’s largest seagrass resources. To provide a foundation to monitor, establish trends and manage the protection of seagrass meadows in the GBRWHA we quantified potential seagrass community extent using six random forest models that include environmental data and seagrass sampling history. We identified 88,331 km^2^ of potential seagrass habitat in intertidal and subtidal areas: 1111 km^2^ in estuaries, 16,276 km^2^ in coastal areas, and 70,934 km^2^ in reef areas. Thirty-six seagrass community types were defined by species assemblages within these habitat types using multivariate regression tree models. We show that the structure, location and distribution of the seagrass communities is the result of complex environmental interactions. These environmental conditions include depth, tidal exposure, latitude, current speed, benthic light, proportion of mud in the sediment, water type, water temperature, salinity, and wind speed. Our analysis will underpin spatial planning, can be used in the design of monitoring programs to represent the diversity of seagrass communities and will facilitate our understanding of environmental risk to these habitats.

## Introduction

Coastal marine habitats are some of the most at-risk ecosystems in the world^1^. Proximity to land-based anthropogenic activities exposes these habitats to threats from multiple stressors^2^. The scale and complexity of marine habitats and the high cost of sampling them means the data used to inform management is often less precise than for equivalent terrestrial systems^3^. This is compounded by significant gaps in the data available for important risks and threats, asymmetry in ecological connectivity, a lack of long-term historical data, enormous variations in scale and poorly documented temporal cycles of impacts and recovery^4–6^. It is difficult to detect even large changes in status and distribution for some coastal and marine habitats, with the current extent and consistency of spatial coverage of monitoring.

Understanding the factors that support the resilience of important coastal marine habitats at large regional scales is difficult. Challenges include describing diversity, distribution and connectivity within ecosystems, defining desired state and assessing ecosystem condition to understand long-term trends and in evaluating short-term impact-recovery cycles^7^. This is exacerbated in Australia’s Great Barrier Reef World Heritage Area (GBRWHA) by the vaguely defined objective and high bar set by the reef management authority in 2015 to “maintain diversity of species and ecological habitats in at least a good condition and with a stable to improving trend”^8^ and updated in 2018 to “[facilitate] adaptive management for the Reef that is effective, efficient and evolving”^9^. Compounding these issues in the GBRWHA is the difficulty of developing and implementing appropriate management frameworks to maintain resilience within multiple priority habitats within the time and investment constraints typically faced by marine management agencies.

Spatial data and visualization techniques are important in understanding and communicating options for managing large and complex coastal marine habitats. Habitat and community maps are a frequently used spatial tool that visualise and assist in evaluation of the association of species and communities of interest with key environmental drivers likely to affect those communities^10^. The ability of these maps to capture the diversity of environmental features that support biological complexity provides the foundation for large-scale spatial assessments of where habitats and communities are likely located^11^. Spatial maps also support an analysis of connectivity^12^, an understanding of spatial and temporal change^13^, and an analysis that defines a desired state^7^. They are a critical component of marine spatial planning, to resolve conflicts, incorporate indigenous knowledge, define management units, and to design representative monitoring programs^14–17^.

Seagrasses form one of the most extensive and important marine coastal habitats in the world, with a diversity that includes 72 species in six families that frequently co-occur as combinations of species or communities^18–21^. Seagrass communities grow in diverse locations in the GBRWHA, including estuaries, lagoons, reef-tops and open seas, intertidal through to deep subtidal, in tropical and temperate regions, and across gradients in water temperature, salinity, desiccation, bottom current stress, light and water quality^18,22,23^. Services provided by these seagrass meadows include coastal protection, food and shelter for fish and crustaceans, nutrient cycling, particle trapping, and removal of bacterial pathogens^24–27^. Seagrass meadows also support populations of charismatic mega herbivores including dugongs (*Dugong dugon*) and green sea turtles (*Chelonia mydas*)^28,29^ and are a valuable marine carbon sink^30^. Within the GBRWHA seagrass meadows can be found range from tropical (10° S) to subtropical (~ 25° S)^4^, and from estuaries to the edge of the outer barrier reef. They also extend north and south of GBRWHA boundaries into the Torres Strait^31^ and south-east Queensland^32,33^.

Seagrasses in the GBRWHA are vulnerable to a range of threats, particularly those associated with declines in water quality and available light, which can have catastrophic consequences^34,35^. Watershed-derived pollutants, particularly sediment loads, were linked to seagrass loss across the GBRWHA from 2008 to 2011^4,36^. Seagrasses in the region also are vulnerable to localized disturbances such as those associated with ports and coastal developments^37,38^. Large-scale and local events have focused research on the response and resilience of seagrass ecosystems to disturbance^39,40^.

Four broad classifications have been applied to describe seagrasses in the GBRWHA: estuarine, coastal, deep-water (subtidal), and reef, and the dominant environmental factors influencing seagrasses identified as terrigenous runoff, physical disturbance, low light, and low nutrients^41,42^. Seagrass communities within each of these four classes are diverse and complex^7^. Previous GBRWHA-scale seagrass spatial models have focussed on overall seagrass distribution or on the distribution of single species. These models were limited by data availability to specific regions (e.g., coastal, deep-water) and/or were at a spatial scale (e.g., ≥ 1 km grids) too large to capture the smaller-scale (metres) areas of seagrass such as narrow intertidal bands within estuaries, or were part of much larger modelling projects that excluded the possibility of using detailed GBRWHA-specific environmental data^18,22,43^.

Recent seagrass data consolidation and greatly improved biophysical environmental data layers provide an opportunity to classify seagrass habitat in more detail than previously possible and, for the first time, to describe seagrass community types throughout the GBRWHA. There is now available a compilation of 35 years of seagrass surveys^44^, and spatially-resolved GBRWHA-wide spatial models of environmental conditions available for depth^45^, tidal exposure^46,47^, hydrodynamics^48^, benthic light^49,50^ and sediment^49,51^.

Our objective in this paper is to use this new information to quantify seagrass and seagrass community structuring at the GBRWHA-scale by: (1) identifying potential seagrass habitat within the GBRWHA and the environmental conditions that underpin the presence or absence of seagrass habitat; and (2) classifying the diverse communities within seagrass habitats on the basis of species diversity and environmental conditions. This analysis will inform decisions for more precise marine spatial planning, management, monitoring, evaluating and mitigating risk, and restoration of seagrasses.

## Results

### Seagrass habitat

**Figure 1:**
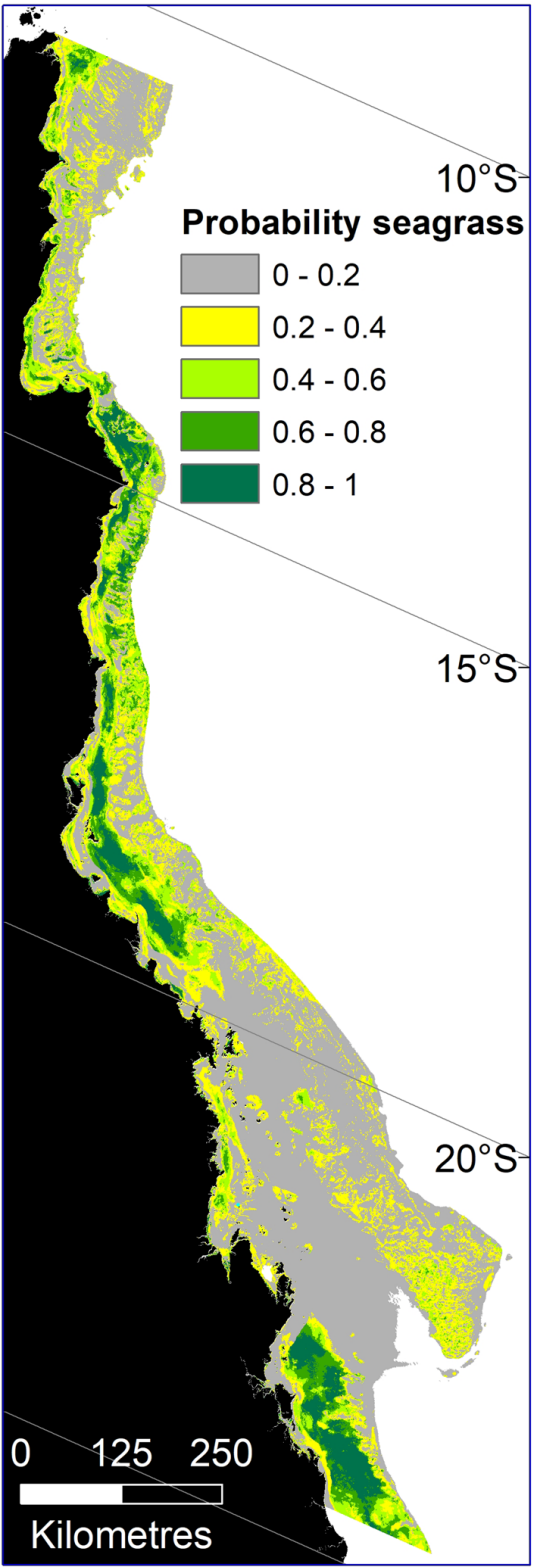
Predicted probability of seagrass presence across the Great Barrier Reef World Heritage Area and adjacent estuaries based on six Random Forest models. Potential seagrass habitat classed as probability ≥ 0.2 (calculated as the average value over the predictions of all the trees in the forest). Map created using ArcGIS software version 10.8 by Esri (www.esri.com).

Using random forest (RF) statistical models we identified approximately 88,321 km^2^ of potential seagrass habitat (probability of seagrass present ≥ 0.2) in the GBRWHA (Fig. 1) as a function of 12 environmental variables. This includes 1111 km^2^ of potential seagrass habitat in estuaries, 16,276 km^2^ in coastal areas, and 70,934 km^2^ in reef areas (Table 1). The performance of RF models varied. Estuary subtidal and intertidal models were the least accurate (72 and 73% overall accuracy, respectively) and reef subtidal and intertidal models were the most accurate (81 and 84% overall accuracy, respectively) (see ESM Appendix S1 in “Supporting Information”).

**Table 1:** Potential seagrass habitat (km^2^) for each probability class across the Great Barrier Reef World Heritage Area and adjacent estuaries based on six Random Forest models.

Probability of seagrass	Model						Total
	Estuary		Coast		Reef		
	Intertidal	Subtidal	Intertidal	Subtidal	Intertidal	Subtidal	
< 0.2	125	473	124	17,829	3,070	79,306	100,927
0.2–< 0.4	99	203	319	9466	820	29,893	40,800
0.4–< 0.6	196	58	323	4006	594	16,419	21,596
0.6–< 0.8	116	49	110	1487	269	12,075	14,106
≥0.8	197	193	56	509	141	10,723	11,819
Total (0.2–1.0)	608	503	808	15,468	1,824	69,110	88,321

The importance of different environmental variables in predicting seagrass presence differed among the six RF models (Table 2). In subtidal areas, depth was the most important environmental condition in estuaries and coasts, and the second most important after benthic light (PARb) in reef areas. The least important of the environmental conditions included for predicting seagrass habitat in subtidal coastal and reef areas was water type, and in estuaries dominant sediment type.

**Table 2:** Importance of environmental variables for each Random Forest model.

Environmental variable	Model					
	Estuary		Coast		Reef	
	Intertidal	Subtidal	Intertidal	Subtidal	Intertidal	Subtidal
Current speed	–	–	51	94	–	41
Depth	–	**215**	–	**159**	–	54
Geomorphology	–	–	–	32	27	–
Latitude	104	162	–	–	–	–
PARb	–	–	63	94	28	**57**
Proportion mud	–	–	54	92	41	49
Salinity	–	–	69	103	–	–
Sediment type	70	92	–	–	–	–
Tidal exposure	**108**	–	63	–	21	–
Water temperature	–	–	70	98	**51**	53
Water type	–	–	24	23	28	31
Wind speed	–	–	**71**	103	40	42

Values are the mean decrease of accuracy in predictions on the out-of-bag samples when a given variable is excluded from the model. The most important variable is in bold.

“–” indicates variable not included in model.

In intertidal areas of estuaries, relative tidal exposure was the most important environmental condition for predicting seagrass presence. In contrast, on reefs, tidal exposure was least important and water temperature was most important. For coastal intertidal areas, wind speed was most important, followed by water temperature, salinity, tidal exposure and benthic light (Table 2).

**Figure 2:**
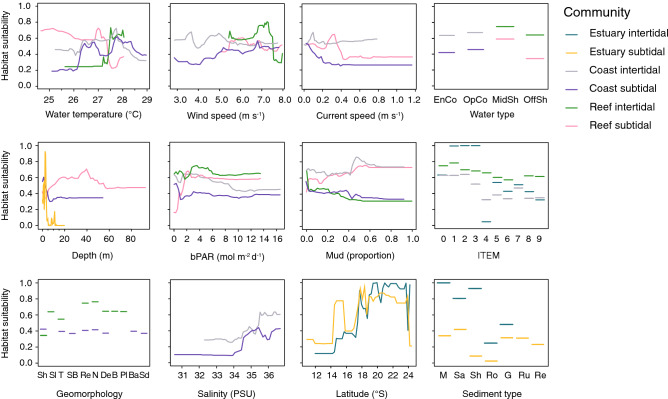
Partial plots of variable importance from six Random Forest models. Abbreviations for factor levels are: Water type (*EnCo* enclosed coastal, *OpCo* open coastal, *MidSh* midshelf; *OffSh* offshore); Geomorphology (*Sh* shelf, *Sl* slope, *T* terrace, *SB* sand bank, *Re* reef, *N* N/A beyond the extent of the layer or on land, *De* deep hole or valley, *B* basin, *Pl* plateau, *Ba* basin, *Sd* saddle); and Sediment (*M* mud, *Sa* sand, *Sh* shell, *Ro* rock, *Ru* rubble, *Re* reef).

The relationship between each environmental predictor and the probability of seagrass being present varied among the models (Fig. 2). In subtidal areas in estuaries, the probability of seagrass presence declines in the first 5 m to p < 0.2. In coasts, the probability of seagrass presence reduces over the first 10 m and then stabilises at p ~ 0.35, while in reefs the probability of seagrass increases between 0 and 40 m depth, then declines between 40 and 60 m to p ~ 0.45 (Fig. 2). In coastal and reef intertidal areas, there was a positive relationship between the proportion of mud in the sediment and probability of seagrass, while in coastal and reef subtidal areas, it was a slightly negative relationship (Fig. 2). In reef areas, there was a greater extent of potential seagrass habitat in subtidal than in intertidal areas, while in coastal areas the extent of potential seagrass was greater in the intertidal zone (Fig. 2).

There were distinct environmental thresholds identified by some models. In reef areas, the long-term annual average temperature of ~ 27 °C was a threshold. Above that temperature, seagrass probability decreased in intertidal areas but increased in subtidal areas (Fig. 2). In both intertidal and subtidal coastal areas, the probability of seagrass increased with water temperature > 26 °C, then declined once waters were > 28 °C (Fig. 2). The probability of seagrass was always greatest where current speeds were lowest and salinity was > 34 PSU. Latitude had a strong effect on the probability of intertidal and subtidal estuarine seagrass, which was most likely to be present at latitudes > 18° S (Fig. 2).

### Seagrass communities

**Figure 3:**
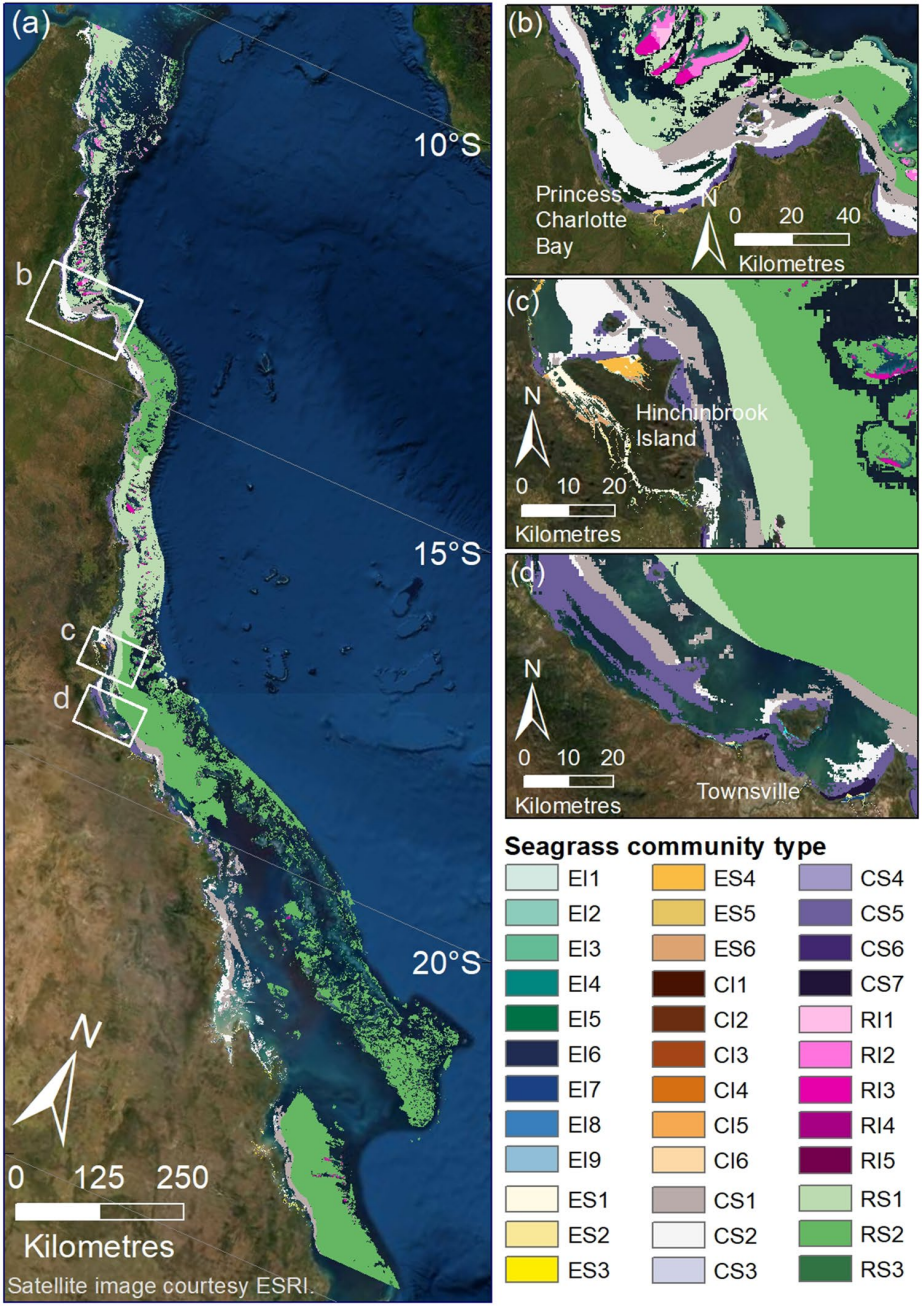
(**a**) Thirty-six seagrass communities predicted for the Great Barrier Reef World Heritage Area and adjacent estuaries: estuary intertidal (EI1–EI9), estuary subtidal (ES1–ES6), coastal intertidal (CI1–CI6), coastal subtidal (CS1–CS7), reef intertidal (RI1–RI5), and reef subtidal (RS1–RS3) communities. (**b**–**d**) Finer-scale maps demonstrating predicted boundaries between communities at select locations. Map created using ArcGIS software version 10.8 by Esri (www.esri.com). Satellite image copyright Esri.

**Figure 4:**
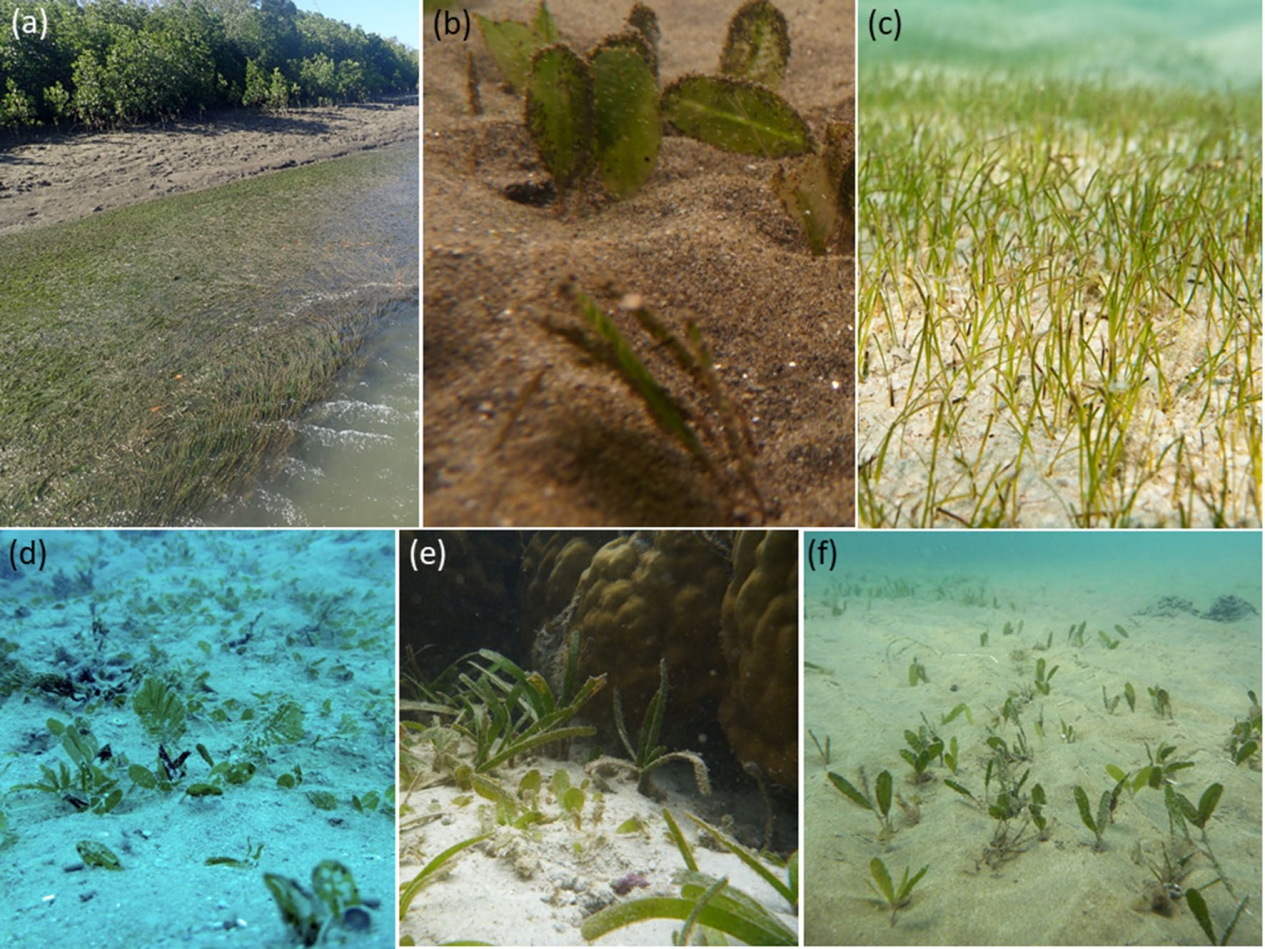
Common seagrass communities in the Great Barrier Reef World Heritage Area and adjacent estuaries: (**a**) estuary intertidal *Z. capricorni*, (**b**) estuary subtidal *H. ovalis* dominated, (**c**) coastal intertidal *H. uninervis* dominated, (**d**) coastal subtidal *H. ovalis* and *H. spinulosa*, (**e**) reef intertidal *T. hemprichii* and *H. ovalis*, and (**f**) reef subtidal *H. decipiens*. Photo credit: TropWATER, James Cook University.

**Figure 5:**
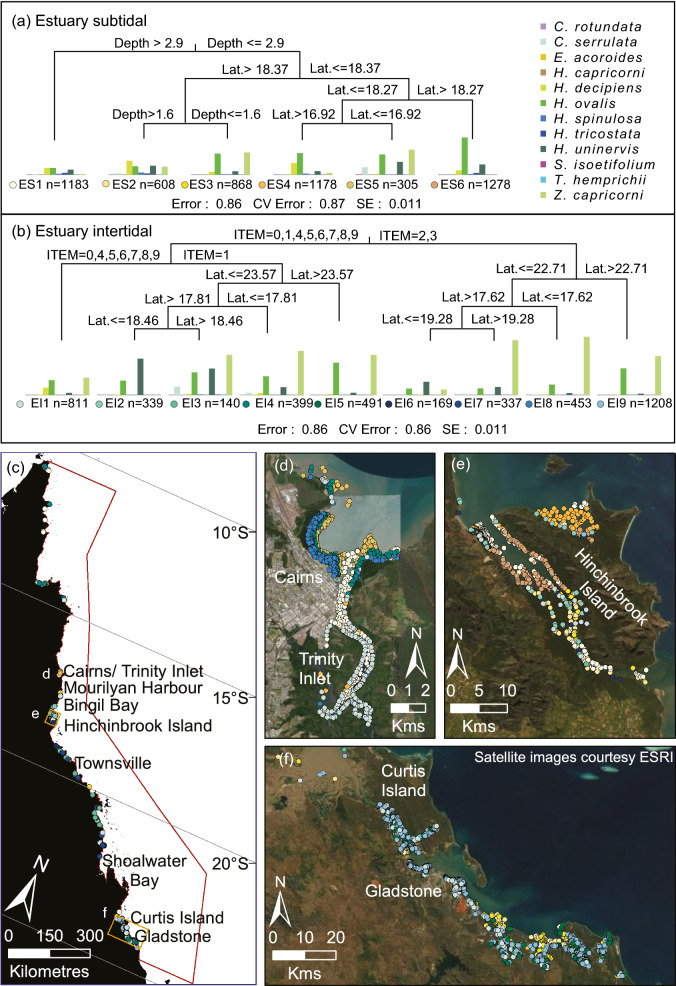
Multivariate regression tree (MRT) and seagrass communities classified for estuaries using species presence/absence data for (**a**) subtidal sites and (**b**) intertidal sites. The number (n) below each community is the count of observations that fall into that community. The histogram shows the frequency of occurrence for each species in that community with the height of the bar representing the frequency that each species was observed in that assemblage. The coloured dots represent unique communities for coast intertidal (EI) 1–9, and coast subtidal (ES) 1–6. The CV Error is the cross-validated relative error. (c) The spatial distribution of communities across the Great Barrier Reef World Heritage Area (red border), and (d-f) finer-scale maps of communities at select locations. Map created using ArcGIS software version 10.8 by Esri (www.esri.com). Satellite image copyright Esri.

**Figure 6:**
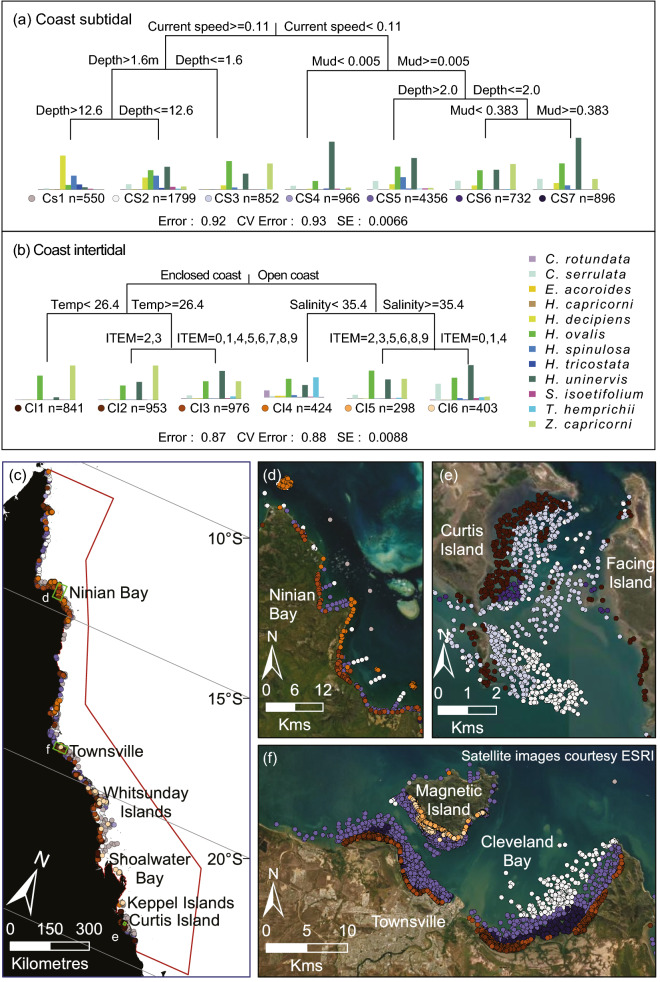
Multivariate regression tree (MRT) and seagrass communities classified for coastal waters using species presence/absence data for (**a**) subtidal sites and (**b**) intertidal sites. The number (n) below each community is the count of observations that fall into that community. The histogram shows the frequency of occurrence for each species in that community with the height of the bar representing the frequency that each species was observed in that assemblage. The coloured dots represent unique communities for coast intertidal (CI) 1–6, and coast subtidal (CS) 1–7. The CV Error is the cross-validated relative error. (**c**) The spatial distribution of communities across the Great Barrier Reef World Heritage Area (red border), and (**d**–**f**) finer-scale maps of communities at select locations. Map created using ArcGIS software version 10.8 by Esri (www.esri.com). Satellite image copyright Esri.

**Figure 7:**
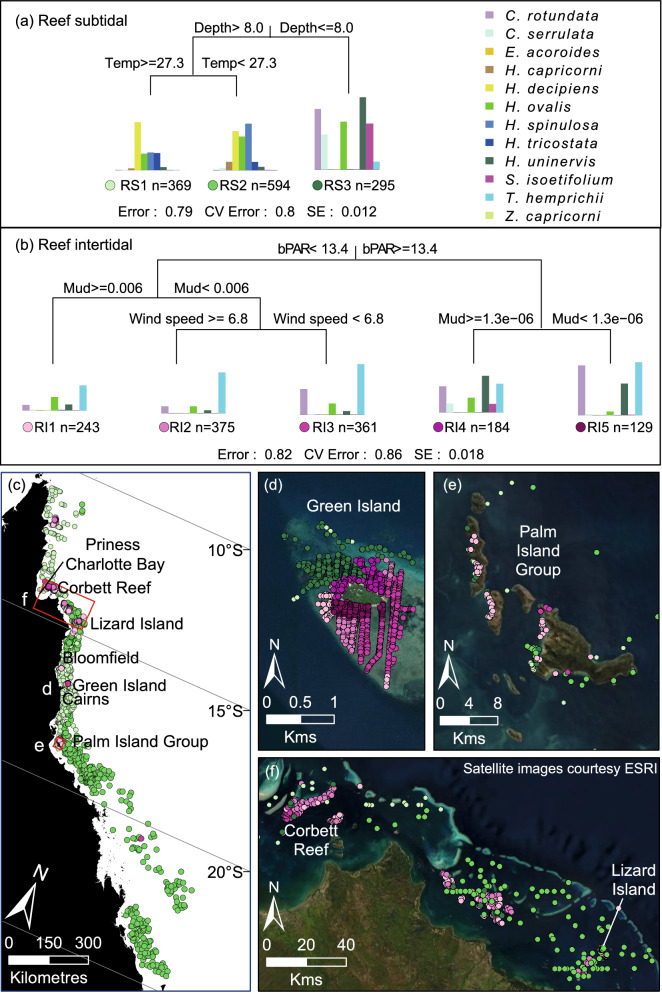
Multivariate regression tree (MRT) and seagrass communities classified for reef waters using species presence/absence data for (**a**) subtidal sites and (**b**) intertidal sites. The number (n) below each community is the count of observations that fall into that community. The histogram shows the frequency of occurrence for each species in that community with the height of the bar representing the frequency that each species was observed in that assemblage. The coloured dots represent unique communities for reef intertidal (RI) 1–5, and reef subtidal (RS) 1–3. The CV Error is the cross-validated relative error. (**c**) The spatial distribution of communities across the Great Barrier Reef World Heritage Area (red border), and (**d**–**f**) finer-scale maps of communities at select locations. Map created using ArcGIS software version 10.8 by Esri (www.esri.com). Satellite image copyright Esri.

Within regions of potential seagrass habitat, we identified 36 seagrass community types defined by their species assemblages (Figs. 3 and 4, Table 3), based on the results of Multivariate Regression Trees (MRTs). The importance of environmental conditions in structuring the location and spatial extent of these communities also was diverse. Important variables included depth, tidal exposure, latitude, current speed, benthic light, proportion of mud in the sediment, water type, water temperature, salinity and wind speed (Figs. 5, 6, 7).

**Table 3:** Seagrass communities in the Great Barrier Reef World Heritage Area and adjacent estuaries, including predicted area and geographic range. See Figs. 5, 6 and 7 for locations.

Community	Predicted area (km^2^)	Geographic range
Estuary Intertidal 1	288	Northern to southern extent GBRWHA
Estuary Intertidal 2	5	South of Bingil Bay to southern end Hinchinbrook Island
Estuary Intertidal 3	77	Southern end Hinchinbrook Island to northern tip Curtis Island
Estuary Intertidal 4	3	Northern extent of GBRWHA to Bingil Bay
Estuary Intertidal 5	7	Northern tip Curtis Island to southern extent GBRWHA
Estuary Intertidal 6	4	South of Mourilyan Harbour to Townsville
Estuary Intertidal 7	156	South of Townsville to Shoalwater Bay
Estuary Intertidal 8	5	Northern extent of GBRWHA to Mourilyan Harbour
Estuary Intertidal 9	39	South of Shoalwater to southern extent GBRWHA
Estuary Subtidal 1	182	Northern to southern extent GBRWHA
Estuary Subtidal 2	96	Hinchinbrook Island to Gladstone
Estuary Subtidal 3	122	Hinchinbrook Island to Gladstone
Estuary Subtidal 4	36	Northern Hinchinbrook Island and the upper reaches of Trinity Inlet
Estuary Subtidal 5	38	Cairns to northern extent of GBRWHA
Estuary Subtidal 6	16	Central and northern Hinchinbrook Island
Coastal Intertidal 1	141	Whitsunday Islands to southern extent GBRWHA
Coastal Intertidal 2	91	Northern to southern extent GBRWHA
Coastal Intertidal 3	205	Northern to southern extent GBRWHA
Coastal Intertidal 4	178	Northern to southern extent GBRWHA
Coastal Intertidal 5	39	Townsville to southern extent GBRWHA
Coastal Intertidal 6	154	Whitsunday Islands to southern extent GBRWHA
Coastal Subtidal 1	7589	Northern to southern extent GBRWHA
Coastal Subtidal 2	4575	Northern to southern extent GBRWHA
Coastal Subtidal 3	68	Northern to southern extent GBRWHA
Coastal Subtidal 4	161	Northern to southern extent GBRWHA
Coastal Subtidal 5	2938	Northern extent GBRWHA to Whitsunday Islands
Coastal Subtidal 6	62	Northern to southern extent GBRWHA
Coastal Subtidal 7	75	Northern to southern extent GBRWHA
Reef Intertidal 1	318	Northern to southern extent GBRWHA
Reef Intertidal 2	887	Northern to southern extent GBRWHA
Reef Intertidal 3	608	Northern to southern extent GBRWHA
Reef Intertidal 4	9	Small reef patches northern to southern extent GBRWHA
Reef Intertidal 5	1	Small reef patches in Cairns and Princess Charlotte Bay regions
Reef Subtidal 1	19,434	Northern extent GBRWHA to Princess Charlotte Bay; Bloomfield to Palm Island Group
Reef Subtidal 2	49,052	Princess Charlotte Bay to Bloomfield; Palm Island Group to southern extent GBRWHA
Reef Subtidal 3	623	Northern to southern extent GBRWHA

Estuaries have communities with the smallest spatial extent in the GBRWHA. Five estuarine communities had a predicted total area between 4 and 7 km^2^ (Table 3). Estuary communities were predicted by variations in relative tidal exposure, depth and latitude, but not the dominant sediment type (Fig. 5). Hinchinbrook Island in the central GBRWHA was identified as an area of high community diversity and a transition zone between communities for both intertidal and subtidal estuarine communities (Figs. 3 and 5e; Table 3).

Coastal communities occur in a highly dynamic zone between estuaries and reefs. Coastal seagrass communities were predicted by the greatest variety of environmental variables and were the only area where all 12 seagrass species were present (Fig. 6).

Reef communities have a distinct species composition. Species such as *Thalassia hemprichii*,* Cymodocea rotundata* and Sy*ringodium isoetifolium* often dominate intertidal and shallow subtidal reef communities, while species found in estuarine and coastal habitats (*Enhalus acoroides* and *Zostera capricorni*) were not present.

### Estuary intertidal

Nine seagrass communities were predicted in the estuarine intertidal model (Figs. 3 and 5, Table 3). *Z. capricorni*, *Halodule uninervis* and *Halophila ovalis* were found in nearly all of these communities with varying frequencies of occurrence (Figs. 4a and 5). The most extensive estuarine intertidal community, EI1, was predicted to cover a total ~ 288 km^2^ throughout the GBRWHA in areas associated with extremely infrequent (intertidal extent model (ITEM) = 0) and medium to high tidal exposure (ITEM = 4–9). The remaining estuarine intertidal communities were predicted to occur in distinct latitudinal bands. Four intertidal communities occurred where tidal exposure was very low (ITEM = 1): the *Z. capricorni* dominated community EI4 in the northern GBRWHA, the *H. uninervis* dominated community EI2 between Bingil Bay and Hinchinbrook Island (17.81°–18.46° S), the mixed species community EI3 between Hinchinbrook Island and northern Curtis Island (23.57° S), and the *H. ovalis* and *Z. capricorni* dominated community EI5 from Curtis Island south (Figs. 3 and 5f; Table 3). An additional four intertidal communities were predicted where tidal exposure was low (ITEM = 2–3): the *Z. capricorni* dominated community EI8 north of Mourilyan Harbour, the *H. uninervis* dominated community EI6 between Mourilyan Harbour and Townsville (17.62°–19.28° S), the extensive *Z. capricorni* dominated community EI7 between Townsville and Shoalwater Bay (156 km^2^), and the *Z. capricorni* and *H. uninervis* dominated community EI9 south of Shoalwater (19.28° S) (Figs. 3 and 5; Table 3).

### Estuary subtidal

The estuary subtidal model predicted six seagrass communities (Figs. 3 and 5; Table 3). Community ES1 is the most extensive, predicted to cover a total subtidal area of 182 km^2^ in depths below 2.9 m mean sea level (MSL). This is the only deep estuarine subtidal community predicted to occur, and it is dominated by *H. ovalis* and/or *Halophila decipiens* with no *Z. capricorni* (Figs. 4b and 5). Other subtidal communities occur in depths shallower than 2.9 m (Fig. 5e). Between Hinchinbrook Island (18.37° S) and Gladstone, community ES2 is predicted in the intermediate depth range 1.6–2.9 m MSL and has a species mix similar to the deep estuarine community ES1 but with the addition of *Z. capricorni*, and community ES3 is predicted in the 0–1.6 m MSL depth range with *Z. capricorni* and *H. ovalis*. From Hinchinbrook Island north, subtidal communities were predicted to occur in distinct latitudinal bands similar to intertidal communities: the small *H. ovalis* community ES6 (16 km^2^) between central and northern Hinchinbrook Island (18.37°–18.27° S), the *H. ovalis*/ *H. decipiens* community between northern Hinchinbrook Island and Trinity Inlet, and the mixed species community ES5 north of Trinity Inlet (16.92° S) (Figs. 3 and 5, Table 3).

### Coastal intertidal

The coastal intertidal model predicted communities separated by variations in water type, water temperature, salinity and tidal exposure (Fig. 6). Three communities were predicted within the enclosed coastal water type: in cooler (< 26.4 °C) southern GBRWHA waters the *H. ovalis* and *Z. capricorni* dominated community CI1; in warmer waters the *Z. capricorni* community CI2 where tidal exposure is low (ITEM = 2–3) and the more speciose community CI3 where tidal exposure is very low (ITEM = 0–1) or intermediate to high (ITEM = 4–9) (Figs. 3 and 6, Table 3). Three communities were also predicted within the open coastal water type. Community CI4 is predicted to occur throughout the GBRWHA where salinity is < 35.4 PSU and, unusually for coastal communities, this speciose community has relatively high frequency of *T. hemprichii* and *C. rotundata,* species usually associated with intertidal reef communities. Communities CI5 and CI6 were predicted to occur in regions of high salinity between Townsville and the Keppel Islands: Community CI5 in areas of low (ITEM = 2–3) and high (ITEM ≥ 5) tidal exposure and community CI6 in areas of very low (ITEM = 0–1) and intermediate (ITEM = 4) tidal exposure (Figs. 3, 4c and 6, Table 3).

### Coastal subtidal

The coastal subtidal model predicted communities separated by variations in current speed, depth, and the proportion of mud in the sediment (Fig. 6). Four communities were associated with very low current speeds (< 0.11 ms^−1^): the *H. uninervis* dominated community CS4 in areas where almost no mud (proportion mud < 0.005) is present in the sediment, and the more diverse communities CS5, CS6 and CS7 when some mud is present. Community CS5 is the largest of these low current communities (2938 km^2^) and predicted at depths > 2 m MSL from the Whitsunday Islands north (Fig. 3), with ten species recorded. Communities CS6 and CS7 are predicted to occur throughout the GBRWHA at depths < 2.0 m: CS6 where the proportion of mud is low to moderate (0.005–0.38), and CS7 where the proportion of mud is > 0.38 and the frequency of *H. uninervis* and *Halophila spinulosa* is greater than in community CS6 (Fig. 6).

Three coastal subtidal communities were predicted to occur throughout the GBRWHA where current speed was > 0.11 ms^−1^. The predicted area of these communities was much larger than low current communities, and communities were associated with different depths. Community CS3 in shallow subtidal waters (< 1.6 m MSL) had a species mix similar to coastal intertidal communities. The large (4575 km^2^) community CS2 at intermediate depths (1.6–12.6 m MSL) was dominated by *H. uninervis* and *H. ovalis* but with a much greater prevalence of typical subtidal species such as *H. decipiens*, *H. spinulosa*, and *Cymodocea serrulata*, and very little *Z. capricorni*. The deep subtidal community CS1 (> 12.6 m) had the largest predicted total area (7589 km^2^) of all coastal communities. This community was dominated almost entirely by *H. decipiens* and *H. spinulosa*, and was one of the few seagrass communities where *Halophila tricostata*, an endemic seagrass species is present (Figs. 3, 4d and 6, Table 3).

### Reef intertidal

Intertidal reef communities were best predicted by a model that included benthic light, proportion mud, and wind speed (Fig. 7). Three reef intertidal communities were associated with light levels < 13.4 mol photons m^−2^day^−1^. *T. hemprichii* was the dominant species in all of these communities (Fig. 4e). *H. ovalis* occurred in greatest frequency in community RI1, predicted to be most prevalent on fringing reefs around the Palm Island Group in the central GBRWHA and as small patches on reefs north of there when some mud is present in the sediment (Figs. 3, 7, Table 3). The large intertidal communities RI2 (887 km^2^) and RI3 (608 km^2^) were associated with very low mud content: RI2 in the northern GBRWHA where wind speed was high (> 6.8 ms^−1^) and RI3 throughout the GBRWHA in calmer conditions (Fig. 7). Communities RI4 and RI5 were associated with high light levels (> 13.4 mol photons m^−2^ day^−1^; Fig. 7). Both communities were characterised by similar frequencies of the dominant species *T. hemprichii, C. rotundata* and *H. uninervis*, but variations in other species depended on the proportion of mud in the sediment with greater species diversity in community RI4 with the addition of mud. Communities RI4 and RI5 were predicted to occur as small patches on reef tops mostly confined to clusters of reefs near Cairns and Princess Charlotte Bay (Fig. 3, Table 3).

### Reef subtidal

The reef subtidal model predicted three reef communities separated by depth and water temperature (Fig. 7). Community RS3 was found at depths < 8 m MSL in the transition zone between intertidal and deep subtidal reef communities. This community was predicted to occur as narrow perimeter bands around reefs and islands throughout the GBRWHA, but particularly on reefs between the Palm Island Group and Bloomfield, and on nearshore reefs north of Princess Charlotte Bay (Figs. 3 and 7, Table 3). Species composition for RS3 was similar to the intertidal reef communities RI4 and RI5: *C. rotundata*, *H. ovalis* and *H. uninervis* frequently occur, but the dominant intertidal species *T. hemprichii* was replaced by *C. serrulata* and *S. isoetifolium* (Fig. 7).

The two largest seagrass communities were associated with reef waters > 8 m MSL (Fig. 7). Both deep communities were dominated by a mix of *Halophila* species, but the frequency of each species varied with water temperature. Community RS1 (19,434 km^2^) was predicted in warmer waters (> 27.3 °C) north of the Palm Island Group, was dominated by *H. decipiens*, and the relatively uncommon *H. tricostata* which was found in this community more often than in any other. The cooler-water subtidal community RS2 (49,052 km^2^) was predicted south of the Palm Island Group and around a cool-water area in the Lizard Island region of the northern GBRWHA (Figs. 3 and 7). Community RS2 is characterised by a more even mix of *Halophila* species: *H. decipiens*, *H. ovalis*, and *H. spinulosa* are all common. The rarer species *Halophila capricorni* is found in this community more often than in any other (Figs. 4f and 7, Table 3).

## Discussion

We present an approach to define seagrass habitat and communities and how they are distributed over large spatial scales. Our study area is vast and encompasses a multitude of changing physical and biological conditions as well as diverse seagrass species. Despite these challenges (and a dataset sourced from multiple studies that collected observations at different times and spatial scales), our approach provides a statistically valid and transferrable approach for one of the world’s most complex seagrass systems. This approach could also be adapted for use at other locations to identify the seagrass community types that make up the seagrass biome, addressing the critical gaps in spatial knowledge needed for global seagrass management and protection^10,52,53^. Our seagrass community model provides a spatial tool needed for understanding seagrass community distribution; a critical pre-requisite for assessing connectivity, resilience, dispersal and restoration decisions^54^.

The advantage of constrained clustering techniques, such as the MRTs we applied to define community types, is that each cluster defines an assemblage type, but additionally the environmental values define an associated habitat type for the assemblage. This allowed prediction of assemblage types where the set of environmental values were available but there was no seagrass data. Our analysis provides a basis for management authorities to identify likely seagrass communities within environmental management plans that are inadequately protected or exposed to environmental threats^10^.

Creating spatially expansive models at the scale of the GBRWHA constrained the analysis to the environmental data also available at that scale, so our models are unlikely to account for smaller-scale localised differences in seagrass communities and their drivers. For example, our community models demonstrated that very small shifts in depth and tidal exposure can lead to significant shifts in seagrass communities. Depth and tidal exposure were two of the highest resolution environmental data sets we used in our models, but many of the environmental predictors we used represent larger-scale spatial patterns modelled at a 1 km grid scale (e.g., benthic light, wind speed, current speed, water temperature, salinity). This means that variables such as modelled benthic light should be reliable at large-scale seasonal-average spatial patterns, but smaller-scale variations in benthic light that depend on small-scale variations in bathymetric depth and sediment distribution will not be accurately predicted.

Seagrass distribution and communities are shaped by multiple environmental complexities. Large spatial trends were present. Seagrass communities in the northern GBRWHA extend from the coast to the edge of the continental shelf, while in the inshore central region bands where no seagrass was present ran parallel to the coast, and in the south, there are large inter-reef areas with little or no likelihood of seagrasses. Our seagrass habitat model and community classification provide an important tool to make informed decisions at an appropriate scale during marine spatial planning, management, monitoring design, threat mitigation, and habitat restoration. Zoning in the Great Barrier Reef Marine Park (GBRMP) to protect biodiversity and regulate human activities has been in place since 1981 when the region became the world’s first coral reef ecosystem to achieve world heritage status. The GBRMP was rezoned in 2004^55^ and while that represented best practice at the time, rezoning identified only five seagrass bioregions where seagrass was a key element (http://www.gbrmpa.gov.au/__data/assets/pdf_file/0011/17300/nonreef-bioregions-in-the-gbrmp-and-gbrwh.pdf). We now provide those previously missed details of the complexity of seagrass communities, particularly for coastal waters and estuaries, that can be used to inform future management of the GBRWHA. This allows a more nuanced approach to management as management responses and spatial planning decisions can be tailored to take into account the diversity of seagrass habitats.

Estuaries and rivers adjacent to the GBRWHA are small by international standards, but their flow and sediment load variability in a monsoon-influenced coastline makes them both key attributes of the GBRWHA and sources of environmental variability^56^. Our estuarine models highlight the paucity of environmental data for estuaries at this scale—our models were limited to just three environmental variables in estuaries but still predicted 15 communities, with latitude acting as a proxy for other complex environmental conditions.

We identified substantial spatial complexity in community types. Some extend throughout the GBRWHA while some communities are small and localised. We focus on seagrass habitats, but these overlap spatially with other environmental values such as populations of sea turtles and dugong that suggest priority areas for management protection such as the Hinchinbrook Island region where extensive and diverse seagrass communities were predicted.

While we provide a framework to understand spatial patterns in seagrass communities it remains open to management authorities to evaluate a level of concern for protection. Some communities have distinct assemblages, while others are differentiated by only slight changes in relative occurrence of species but have been identified as distinct communities in our analysis because species and environmental features were different. Sensitivity of seagrass communities to environmental threats can also be ameliorated by resilience inferred by connectivity, which is not included in this model but likely to have an influence at scales of hundreds of kilometres^54,57^.

To design a marine protection system for all seagrass communities, these spatial complexities and differing sensitivities to environmental conditions would need to be adapted into a broader marine protection approach. We are now able to better evaluate environmental risk to seagrass habitats from natural processes and anthropogenic activity and to assess environmental threats that affect seagrass at a large scale including cyclones and floods^4,36,58^, climate change^59,60^, and more localised impacts such as coastal development, dredging, and oil spills^61,62^. Spatial assessments of cumulative anthropogenic risk to seagrasses in the GBRWHA found risks tend to accumulate where ports and coastal development pressures overlay with inputs from coastal catchment runoff^38^. Our community model provides a tool to identify communities that occur in these risk hotspots and may be vulnerable due to their lack of representation outside of high-risk areas.

Our classification of seagrass communities provides a spatially comprehensive tool that can be used to assess management actions for seagrass throughout the GBRWHA. The varied environmental conditions that determine seagrass community diversity demonstrate that reporting trends at large scales and with coarse partitions such as “coastal” fail to accurately account for changes at the more precise community level.

Our research emphasizes that the GBRWHA seagrasses do not function as a single entity and reporting of trends will provide a more accurate picture of change if it is reported at the scale of the seagrass communities. We identify communities in locations where data is poor and could be improved by a more comprehensive monitoring program. Our method has potential global utility as an approach to create informative models based on data that is scalable and easily available as it requires only presence/absence data for seagrass species.

## Methods

### Study area

**Figure 8:**
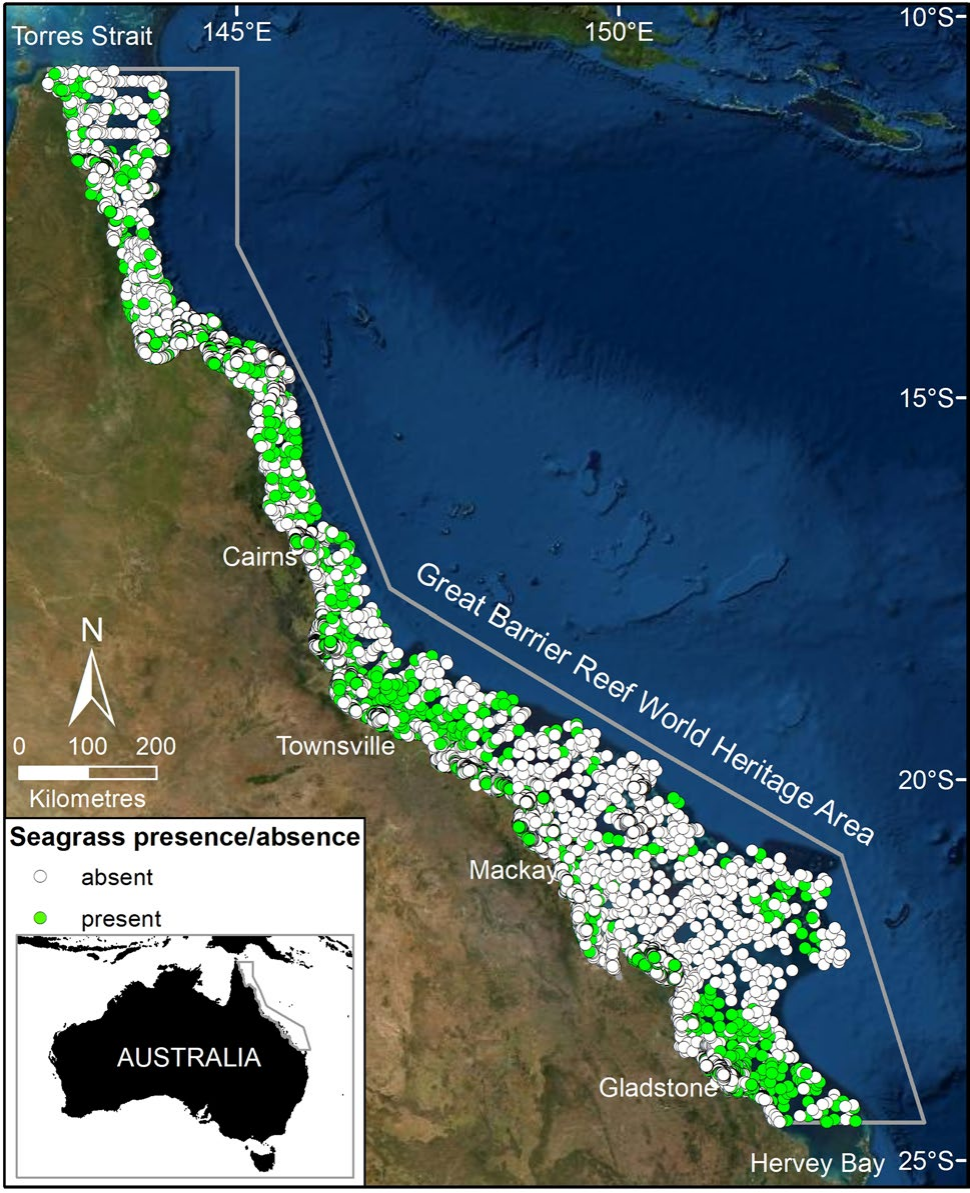
Seagrass presence and absence at sampling sites across the Great Barrier Reef World Heritage Area (grey boundary). Map created using ArcGIS software version 10.8 by Esri (www.esri.com). Satellite image copyright Esri.

The Great Barrier Reef in tropical north-eastern Australia is one the world’s most extensive coral reef structures, an environment home to a globally outstanding and biodiverse marine ecosystem. A Marine Park was proclaimed by the Australian Federal government in 1975 (*Great Barrier Reef Marine Park Act 1975*) and the region was inscribed as a World Heritage Area in 1981. Within the GBRWHA more than 2500 individual reefs and over 900 islands protect an extensive and mostly shallow inter-reef lagoon that extends across the continental shelf. The GBRWHA covers an area of around 350,000 km^2^ in north-east Australia, including 2500 km of coastline and a shelf that extends up to 250 km offshore. Our study area covers the continental shelf region of the GBRWHA and extends into and includes adjacent estuaries along the mainland Australian coast (Fig. 8).

The large size of the GBRWHA means there is large variation in geography, topography, and environmental conditions Fig. 8; ESM Appendix S4^63,64^. The northern GBRWHA (< ~ 16° S) is characterised by a narrow shelf, shallow inter-reef waters (< 30 m), elongate reefs, warmer water temperatures, high benthic light, low current speed, and low salinity (ESM Appendix S4). The central GBRWHA (~ 16° and 20° S) is characterised by lower reef density, intermediate inter-reef depths (> 40 m), low current speed, low salinity and low wind speed (ESM Appendix S4). The southern GBRWHA (> ~ 20°S) is characterised by high reef density areas of deep water (down to 140 m) across a wide continental shelf, high salinity, high current speed, cooler water and lower mud content in the sediment ESM Appendix S4^63,64^. There are also major regional differences along the coast adjacent to the reef lagoon in land type, climate, and land use, e.g., tropical and subtropical, wet and dry tropics, pristine, sugar cane or cattle-dominated catchments^42,65^. Adding to this complexity is a coastal mountain range that in the northern GBRWHA runs close to the coast with mostly small watersheds and short rivers compared with the central and southern GBRWHA. The human population is concentrated in coastal communities of the central and southern coast. Threats and risk to coastal seagrass integrate these broad trends^38^.

### Seagrass data

Seagrass presence/absence data is from a synthesis of seagrass surveys collected throughout the GBRWHA and adjacent estuaries between 1984 and 2018 (ESM Appendix S2). Surveys were conducted for five major purposes: (1) mapping coastal seagrass to ~ 15 m depth in the 1980s; (2) cross-shelf subtidal surveys in the early to mid-1990s and again in 2003–2005; (3) sporadic mapping of intertidal meadows as part of an oil spill response atlas between 2001 and 2014; (4) targeted mapping projects, such as within Dugong Protected Areas; and (5) frequent (at least annual) and spatially intense mapping and monitoring in and adjacent to six Queensland ports, that in some cases extend back more than 25 years. The seagrass data set is publicly available as a single GIS file of 81,387 survey sites (10.25909/y1yk-9w85)^44^. The data includes presence/absence of seagrass (Fig. 8) and each seagrass species, site coordinates, dominant sediment type, and survey month and year. Species included in the data and this analysis are: *C. rotundata* (Ascherson & Schweinfurth, 1870), *C. serrulata* ((R.Brown) Ascherson & Magnus 1870), *E. acoroides* ((Linnaeus f.) Royle, 1839), *H. capricorni* (Larkum, 1995), *H. decipiens* (Ostenfeld, 1902), *H. ovalis* ((R.Brown) J. D. Hooker, 1858), *H. spinulosa* (R.Brown) Ascherson, 1875), *H. tricostata* (Greenway), *H. uninervis* ((Forsskål) Ascherson, 1882), *S. isoetifolium* ((Ascherson) Dandy, 1939), *T. hemprichii* ((Ehrenberg) Ascherson, 1871), and *Z. muelleri subsp. capricorni* ((Ascherson) S. W. L. Jacobs, 2006).

The impact of a series of intense tropical cyclones with high rainfall and flooding that severely reduced seagrass presence and altered species composition along the southern two-thirds of the GBRWHA was quantified by ports long-term monitoring programs between 2009 and 2012 and inshore seagrass monitoring. Recovery has been variable among locations^4,35,58,66^. Previous seagrass community analysis demonstrates species assemblages during and after major disturbance events are disproportionally dominated by colonising species, leading to an overly simplistic community classification relative to the long-term seagrass diversity^7^. Because of this, ports long-term monitoring data was excluded from our analysis if overall seagrass condition was classed as poor or very poor in the annual report card for each of those locations^67–72^. This avoided a bias in the analysis from defining seagrass community types based on data that overwhelmingly represented a significant environmental impact, rather than average environmental conditions. This process was applied only to ports data because these were the only locations in the central and southern GBRWHA where sampling occurred during 2009–2012. Data was also restricted to the seagrass growing season (August–January; included approximately 80% of available site data) to reduce the likelihood of including times and sites in the analysis where seagrass was absent due to the seasonal and ephemeral nature of some species. This is particularly important for deep-water *Halophila* communities, which may be present only as a seed bank through the colder months of the year^37,73^.

### Models and environmental predictors

We fitted random forest and multivariate regression tree models to six subsets of the data: estuary intertidal, estuary subtidal, coast intertidal, coast subtidal, reef intertidal and reef subtidal, each resulting in a different model fit (ESM Appendix S4; Fig. S1a). This separation was used as it accounted for variation in availability of environmental data (e.g. lack of environmental data for estuaries), variation in seagrass sampling history and intensity (e.g. a gradient in sampling intensity that decreases with distance from the Australian mainland coast, and with depth), and well-established general differences in seagrass species distributions (e.g. intertidal versus subtidal species)^7,22^.

For each site we chose spatial data that either directly influenced the category for spatial analysis (e.g. exposure for intertidal habitat and depth for subtidal habitat) or was known from previous studies to influence seagrass distribution and/or community structure (e.g. benthic light). These data sets were used to quantify environmental conditions at each site, and to assign each site to one of the six models (estuarine intertidal, estuarine subtidal, coastal intertidal, coastal subtidal, reef intertidal, reef subtidal) (Table 4; ESM Appendix S2).

**Table 4:** Spatial data used to quantify environmental conditions and determine model boundaries.

Data	Type	Models	Data source
Intertidal/subtidal	Categorical; intertidal, subtidal	All	gbr30 (30 m pixel resolution) raster^45^; ITEM version 2.0^46,47^; tidal regions of reefs or shoals within Queensland maritime waters © State of Queensland (Department of Natural Resources, Mines and Energy) 2019
Depth	Numeric; metres below mean sea level	Subtidal only	gbr30 (30 m pixel resolution) raster^45^
Relative tidal exposure	Categorical; bands 1–9	Intertidal only	Intertidal Extent Model (ITEM version 2.0)^46,47^
Water type	Categorical; estuary, coast, reef	All	Queensland coastal waterways geomorphic habitat mapping estuary boundary^74^. Marine Water Bodies definitions (version 2_4; Data courtesy of the Great Barrier Reef Marine Park Authority)
Sediment	Numeric; proportion mud	Coast and Reef	eReefs 1 km grid hydrodynamic model: https://research.csiro.au/ereefs/models/model-outputs/access-to-raw-model-output/^49,51^
Sediment	Categorical; dominant sediment	Estuary	Great Barrier Reef data synthesis^44^
Benthic geomorphology	Categorical; geomorphic (benthic) features	Coast and Reef	Geomorphic Features of the Australian Margin^75^
Benthic light	Numeric; benthic photosynthetically active radiation (PARb) above the seagrass canopy, mol photons m^−2^ day^−1^	Coast and Reef	“EpiPAR_sg” variable from eReefs 1 km grid biogeochemical and optical model (v924): http://dapds00.nci.org.au/thredds/catalog/fx3/gbr1_bgc_924/catalog.html^49,50^
Water temperature	Numeric (°C)	Coast and Reef	eReefs 1 km grid hydrodynamic model: https://data.aims.ereefs.org.au/thredds/fileServer/derived-download/gbr1_2.0/all-one/all-one.nc^48^
Mean current speed	Numeric (ms^−1^)	Coast and Reef	eReefs 1 km grid hydrodynamic model: https://data.aims.ereefs.org.au/thredds/fileServer/derived-download/gbr1_2.0/all-one/all-one.nc^48^
Salinity	Numeric; Practical Salinity Unit (PSU)	Coast and Reef	eReefs 1 km grid hydrodynamic model: https://data.aims.ereefs.org.au/thredds/fileServer/derived-download/gbr1_2.0/all-one/all-one.nc^48^
Wind speed	Numeric (ms^−1^)	Coast and Reef	eReefs 1 km grid hydrodynamic model: https://data.aims.ereefs.org.au/thredds/fileServer/derived-download/gbr1_2.0/all-one/all-one.nc^48^
Latitude	Numeric	Estuary	ArcGIS

More details are provided in ESM Appendices S3 and S4.

### Statistical analysis

We conducted a two-step analysis to (1) define potential seagrass habitat, then (2) classify seagrass communities within that habitat. To define potential seagrass habitat, we used the machine learning technique random forest (RF) to examine the probability of seagrass occurrence irrespective of species. The RF method is a non-parametric tree-based analysis. It generates multiple classification or regression trees, each calibrated on a bootstrap sample of the original data using a subset of the predictor variables, with the model prediction calculated as the average value over the predictions of all the trees in the forest^76^. The accuracy of the RF model depends on the predictive power of each tree and the correlation between trees^76^.

Random forest models were implemented using the *randomForest* package^77^ in R version 4.0.2^78^. For each RF model, seagrass presence/absence (1/0) data was randomly partitioned into training (80% of data set) and testing (remaining 20%) datasets (Table 5). For each model, we set the number of classification trees (ntree) to 500. The optimal number of predictor variables to be randomly selected at each node (mtry) was determined by tuning each model (Table 5). The importance of predictor variables was assessed using the mean decrease in accuracy. Variables included in each model were plotted using the *plotmo* package^79^ where, for each plot, the background variables are held fixed at their median values (calculated from the training data). Each model was validated using a confusion matrix derived from the independent validation (test) data, using the *caret* package in R^80^. A confusion matrix shows agreement and disagreement in a table format, with predicted values forming the matrix columns and observed values forming the rows. From this matrix we calculated the total accuracy (i.e., percentage of sites correctly classified) and accuracy for each class (present/absent).

**Table 5:** Random Forest (RF) and Multivariate Regression Tree (MRT) model specifications for estuarine, coastal and reef intertidal and subtidal areas in the Great Barrier Reef World Heritage Area and adjacent estuaries.

Model name	RF models		MRT models
	Number of sites	mtry	Number of sites
Estuary intertidal	4962	2	4347
Estuary subtidal	6426	1	5420
Coast intertidal	5328	2	3895
Coast subtidal	16,073	3	10,151
Reef intertidal	2569	2	1292
Reef subtidal	2695	2	1258
Total	38,053	–	26,363

Total number of sites used in each model (split between 80% for model training and 20% for testing), total sites used in each model, and the optimal number of predictor variables that were randomly selected at each node in RF models (mtry).

To avoid the issue of multicollinearity of environmental variables in our models we calculated variance inflation factors (VIF) for all environmental variables. Highly correlated variables (VIF > 3) were removed prior to analysis following the conservative threshold recommended by Zuur et al.^81^: tidal range (collinear with water temperature) was not included in any model; apart from that, collinearity and the variables excluded differed among models. Variables available in the RF models were:

1. $${\text{RF}}_{{({\text{estuary}},\;{\text{intertidal}})}} \sim {\text{Tidal}}\;{\text{exposure}} + {\text{Latitude}} + {\text{Sediment}}$$
2. $${\text{RF}}_{{({\text{estuary}},\;{\text{subtidal}})}} \sim {\text{Depth}} + {\text{Latitude}} + {\text{Sediment}}$$
3. $$\begin{aligned} {\text{RF}}_{{({\text{coast}},\;{\text{intertidal}})}} & \sim {\text{Current}}\;{\text{speed}} + {\text{Tidal}}\;{\text{exposure}} + {\text{PARb}} + {\text{Proportion}}\;{\text{mud}} + {\text{Salinity}} \\ & \quad + {\text{Water}}\;{\text{temperature}} + {\text{Water}}\;{\text{type}} + {\text{Wind}}\;{\text{speed}} \\ \end{aligned}$$
4. $$\begin{aligned} {\text{RF}}_{{({\text{coast}},\;{\text{subtidal}})}} & \sim {\text{Current}}\;{\text{speed}} + {\text{Depth}} + {\text{Geomorphology}} + {\text{PARb}} + {\text{Proportion}}\;{\text{mud}} \\ & \quad + {\text{Salinity}} + {\text{Water}}\;{\text{temperature}} + {\text{Water}}\;{\text{type}} + {\text{Wind}}\;{\text{speed}} \\ \end{aligned}$$
5. $$\begin{aligned} {\text{RF}}_{{({\text{reef}},\;{\text{intertidal}})}} & \sim {\text{Tidal}}\;{\text{exposure}} + {\text{Geomorphology}} + {\text{PARb}} + {\text{Proportion}}\;{\text{mud}} \\ & \quad + {\text{Water}}\;{\text{temperature}} + {\text{Water}}\;{\text{type}} + {\text{Wind}}\;{\text{speed}} \\ \end{aligned}$$
6. $$\begin{aligned} {\text{RF}}_{{({\text{reef}},\;{\text{subtidal}})}} & \sim {\text{Current}}\;{\text{speed}} + {\text{Depth}} + {\text{PARb}} + {\text{Proportion}}\;{\text{mud}} + {\text{Water}}\;{\text{temperature}} \\ & \quad + {\text{Water}}\;{\text{type}} + {\text{Wind}}\;{\text{speed}} \\ \end{aligned}$$

The six RF models were used to generate rasters of seagrass predicted probability across the entire GBRWHA. We created this by predicting each model onto a raster stack of data corresponding to the same predictors included in each model using the *raster* package in R^82^. Raster data sets within each stack were predicted to the 30 m resolution of the depth model^45^ using the *sf* package in R^83^. In this analysis we defined potential seagrass habitat as regions where the RF models predicted a probability ≥ 0.2 rather than a more conservative ≥ 0.5 used previously^22^. This previous level of probability was chosen to identify seagrass distributions for management and zoning advice. In the present analysis it was important to choose a level of probability to be more inclusive and that ensured that no seagrass area was missed in an exercise designed to identify distinct community types. The ≥ 0.2 threshold appropriately captures the extent of seagrass for a communities analysis and avoids excluding areas where seagrass could occur. This threshold still has the effect of excluding from the analysis areas classed as unlikely seagrass habitat and where seagrass has never been previously recorded.

Our second analysis defined seagrass communities within predicted potential seagrass habitat using multivariate regression trees (MRT) in the R package *mvpart*^84^ (available in archive form on CRAN at https://cran.r-project.org). MRT are a constrained analysis that repeatedly splits the assembled data (in this case a matrix of presence/absence data for each species as the response variable for each model) into groups that represent a distinct community composition defined by threshold values of associated environmental variables (De’ath 2002)^85^. Using species presence/absence from each site resulted in the community type being defined based on the frequency of occurrence of each species. For each MRT we used the same environmental predictors as for the RF models. We excluded sites from unlikely seagrass habitat to allow the six MRT models to identify patterns in seagrass species presence without being diluted by zeros due to seagrass absence (Table 5). As the aim was to cluster the sites spatially, we did not include ‘year’ as a factor in the model. Instead, the intent was to categorise where each seagrass species is found, on average, through time.

We selected the best MRT for each habitat model using the cross-validated relative error (CVRE). The CVRE represents the capacity of the tree to predict community composition for new sites. Calculation of the CVRE is based on a repeated random sub-sampling cross-validation, where number of cross-validations can be specified and controls the proportional allocation of sites to training and testing (evaluation) sets and this is repeated ten times, where each time site data are randomly allocated to train and test groups. We designated 80% of our data for model training and 20% for testing. The CVRE is the average test error over the chosen number of cross-validations. We repeated the cross-validation 100 times to stabilise variability in CVRE estimates due to the random cross-validation; the *mvpart* package then estimates the mean CVRE, where 0 indicates perfect prediction and ≥ 1 indicates no predictive power. The depth (number of splits) in the trees was selected by finding that depth that fitted the best predictive tree in the cross-validation.

All maps were created in ArcMap 10.8 (ESRI, Redlands, CA). The area of each seagrass probability level from the RF analysis, and each seagrass community from the MRT analysis, was determined by multiplying the pixel size (900 m^2^) by the total number of pixels for each category of interest in each raster of the modelled predictions for seagrass probability and community type.

## Supplementary Information

Supplementary Information.

## Data Availability

The seagrass site data used in this analysis is available at: https://doi.org/10.25909/y1yk-9w85. The predicted probability of seagrass presence across the Great Barrier Reef World Heritage Area and adjacent estuaries (random forest models) is available at: https://doi.org/10.26274/J6B6-PH79. The predicted distribution of seagrass communities across the Great Barrier Reef World Heritage Area and adjacent estuaries (MRT models) is available at: https://doi.org/10.26274/NRE6-YS16.
