# Supplementary material for: A spatial analysis of seagrass habitat and community diversity in the Great Barrier Reef World Heritage Area
Source: Sci Rep. 2021 Nov 16;11:22344. doi: 10.1038/s41598-021-01471-4 (PMC8595360; doi:10.1038/s41598-021-01471-4)
Supplement: Supplementary file 1 — Supplementary Information. [file 41598_2021_1471_MOESM1_ESM.docx]

# Supporting Information

**Appendix S1.**

Table S1. Random Forest confusion matrices and performance using testing data for six models. Columns show predicted values (P) and rows show observed values (O). Accuracy of model predictions for each class of seagrass absent, present, and overall accuracy.

| RF Model | Confusion Matrix | | | Accuracy (%) |
| --- | --- | --- | --- | --- |
| Estuary Intertidal |  | Absent (O) | Present (O) |  |
|  | Absent (P) | 239 | 95 | 72% (absent) |
|  | Present (P) | 185 | 464 | 72% (present) |
|  |  |  | | 73% (overall) |
| Estuary Subtidal |  | Absent (O) | Present (O) |  |
|  | Absent (P) | 380 | 156 | 71% (absent) |
|  | Present (P) | 194 | 536 | 73% (present) |
|  |  |  | | 72% (overall) |
| Coast Intertidal |  | Absent (O) | Present (O) |  |
|  | Absent (P) | 307 | 108 | 74% (absent) |
|  | Present (P) | 129 | 516 | 80% (present) |
|  |  |  | | 78% (overall) |
| Coast Subtidal |  | Absent (O) | Present (O) |  |
|  | Absent (P) | 1310 | 387 | 77% (absent) |
|  | Present (P) | 391 | 1092 | 74% (present) |
|  |  |  | | 76% (overall) |
| Reef Intertidal |  | Absent (O) | Present (O) |  |
|  | Absent (P) | 257 | 33 | 89% (absent) |
|  | Present (P) | 50 | 166 | 77% (present) |
|  |  |  | | 84% (overall) |
| Reef Subtidal |  | Absent (O) | Present (O) |  |
|  | Absent (P) | 294 | 62 | 83% (absent) |
|  | Present (P) | 39 | 132 | 77% (present) |
|  |  |  | | 81% (overall) |

**Appendix S2.**

Table S2. Survey purpose and location of spatial data used in seagrass data compilation, 1984-2018. Reproduced from Carter et al. (2020).

| **Survey purpose/ data location** | **Year/s** | Reference |
| --- | --- | --- |
| 1. *1980s GBR-scale coastal surveys* | | |
| Cape York to Cairns | 1984, 1985 | (Coles et al., 1985) |
| Cairns to Bowen | 1987 | (Coles et al., 1992) |
| Bowen to Water Park Point | 1987 | (Coles, 1987) |
| Water Park Point to Hervey Bay | 1988 | (Coles et al., 1990) |
| 1. *GBR-scale deep-water surveys* | | |
| GBR Deep-Water | 1994-1999 | (Coles et al., 2009) |
| GBR Seabed Biodiversity | 2003-2005 | (Pitcher et al., 2007) |
| 1. *Oil spill response atlas (OSRA) intertidal surveys* | | |
| Princess Charlotte Bay to Cape Flattery | 2011-2014 | (Carter et al., 2013; Carter et al., 2012; Carter & Rasheed, 2014; Carter & Rasheed, 2015) |
| Hydrographers Passage | 2003 | (Rasheed et al., 2006) |
| Margaret Bay | 2001 | (Rasheed et al., 2005) |
| 1. *Targeted seagrass mapping surveys* | | |
| Bustard Bay | 2009 | (Taylor et al., 2010) |
| Cape Flattery | 1996 | (Ayling et al., 1997) |
| Clairview | 2017-2018 | (Carter & Rasheed, 2019) |
| Clump Point | 1997 | (Roder et al., 1998) |
| Dugong Protection Area | 1999 | (Coles et al., 2002) |
| Dunk Island to Cleveland Bay | 1996 | Unpublished data |
| Edgecumbe Bay | 2008 | (Coles et al., 2007) |
| Green Island | 1997, 2003 | (McKenzie & Lee Long, 1996; McKenzie et al., 2014) |
| Lizard Island | 1995 | (McKenzie et al., 1997) |
| Low Isles | 1997 | (McKenzie et al., 2016) |
| Lucinda to Bowling Green Bay | 2007 | (Coles et al., 2007) |
| Oyster Point | 1995-1998 | (Lee Long et al., 2001) |
| Shoalwater Bay | 1996 | (Lee Long et al., 1996a) |
| Whitsunday Islands | 1999-2000 | (Campbell et al., 2002) |
| 1. *Queensland ports seagrass long-term monitoring surveys* | | |
| Cairns | 1993, 1996, 2000-2018 | (Lee Long et al., 1996b; Rasheed & Roelofs, 1996; Rasheed et al., 2019) |
| Gladstone | 2002-2018 | (Chartrand et al., 2019) |
| Mackay and Hay Point | 2001-2018 | (Rasheed et al., 2001; York & Rasheed, 2019) |
| Abbot Point | 2005-2018 | (McKenna et al., 2019) |
| Mourilyan Harbour | 1993-2018 | (Wells et al., 2019) |
| Townsville | 2007-2018 | (Bryant et al., 2019) |

**Appendix S3.**

Description of spatial data used to determine model boundaries and quantify environmental conditions at each site.

- **Depth** (metres below mean sea level; subtidal sites only) – *numeric data*. Depth below mean sea level (MSL) data for subtidal sites only was extracted from the gbr30 (30 m pixel resolution) raster (Appendix S4; Fig. S1b) (Beaman, 2017). Deep waters extending east of the continental shelf and beyond the historical seagrass data set were excluded (< approx. -100 m). Depth was not included as a variable in intertidal models.
- **Intertidal/subtidal** – *categorical data*. Used to define and to separate data for the intertidal and subtidal models. Sites were classed as intertidal if they fell within the ITEM bands 1–9, or were classed as tidal regions of reefs or shoals within Queensland maritime waters (© State of Queensland (Department of Natural Resources, Mines and Energy) 2019), even where ITEM = 0. This allowed for the inclusion of sites particularly on intertidal reef-tops known from helicopter field surveys to expose during spring tides but that were not defined as intertidal by the ITEM model (Appendix S4; Fig. S1c). All other sites were classed as subtidal.
- **Tidal exposure** – *categorical data*. Relative tidal exposure obtained from the Intertidal Extent Model (ITEM version 2.0) with categorical bands 1–9, where 1 = exposed 0–10% of the time, 9 = exposed 80–100% of the time, and 0 = areas of water across the observed tidal range (Appendix S4; Fig. S1c) (Bishop-Taylor et al., 2019; Geoscience Australia, 2017).
- **Water type** – *categorical data*. Sites were classed as estuarine if they were within the Queensland coastal waterways geomorphic habitat mapping estuary boundary (Dyall et al., 2004). Non-estuarine water types were classified according to the Marine Water Bodies definitions (version 2_4; Data courtesy of the Great Barrier Reef Marine Park Authority): enclosed coastal, open coastal, midshelf and offshore (Appendix S4; Fig. S1d). Values for sites outside these layers were estimated using the nearest water type polygon. These water types were also grouped more broadly to define the estuary, coast (enclosed and open coastal) and reef (midshelf and offshore) models.
- **Sediment** – *numeric data*. For coastal and reef sites the proportion of mud for each site was extracted from the eReefs 1km grid hydrodynamic model (30 January 2018), available at: [https://research.csiro.au/ereefs/models/model-outputs/access-to-raw-model-output/](https://aus01.safelinks.protection.outlook.com/?url=https%3A%2F%2Fresearch.csiro.au%2Fereefs%2Fmodels%2Fmodel-outputs%2Faccess-to-raw-model-output%2F&data=02%7C01%7Calexandra.carter%40jcu.edu.au%7C69ed87af718b46f5bd9608d8637c2cf9%7C30a8c4e81ecd4f148099f73482a7adc0%7C0%7C0%7C637368732305064643&sdata=bOiRHRwl%2Bqp9XZ9RQn7AdjSmvrw0tX0rLsaREF6DXjY%3D&reserved=0) (see also Baird et al., 2020; Margvelashvili et al., 2018) (Appendix S4; Fig. S1e). For enclosed and open coastal sites outside the raster extent, proportion of mud was estimated by inverse distance weighted (IDW) interpolation. For estuarine seagrass, sediment was classified as the dominant sediment type from field descriptions (available in Carter et al., 2021) (categorical data) because the proportion mud raster excluded most estuarine seagrass data, interpolation was not appropriate or the 1km grid size was too coarse for application within the narrow tidal bands of estuaries.
- **Benthic geomorphology** – *categorical data*. Sites were categorised by geomorphic (benthic) features as defined by the Geomorphic Features of the Australian Margin (Appendix S4; Fig. S1f; Heap & Harris, 2008). Benthic geomorphology was excluded from estuary analysis.
- **Benthic light** – *numeric data*. This was extracted from the eReefs 1 km grid biogeochemical and optical model (v924) “EpiPAR_sg” variable, representing benthic photosynthetically active radiation (PARb) above the seagrass canopy in mol photons m^‑2^d^‑1^. These data are available from: <http://dapds00.nci.org.au/thredds/catalog/fx3/gbr1_bgc_924/catalog.html> (see also Baird et al., 2016; Baird et al., 2020). Values are an aggregation of daily benthic light from 2003 to 2019. Data is available for both wet and dry season; we used the dry season data because the two data sets were highly correlated (Appendix S4; Fig. S1g). Values for coastal sites outside the raster extent were estimated by IDW interpolation. Benthic light was excluded from the estuary analysis.
- **Water temperature, mean current speed, and salinity** - *numeric data*. This was extracted from the eReefs 1km grid hydrodynamic model representing water temperature (°C; Appendix S4; Fig. S1h), mean current speed (ms^-1^; Appendix S4; Fig. S1i), and salinity (PSU, Appendix S4; Fig. S1j) at -2.35 m depth below mean sea level, available at: [https://data.aims.ereefs.org.au/thredds/fileServer/derived-download/gbr1_2.0/all-one/all-one.nc](https://aus01.safelinks.protection.outlook.com/?url=https%3A%2F%2Fdata.aims.ereefs.org.au%2Fthredds%2FfileServer%2Fderived-download%2Fgbr1_2.0%2Fall-one%2Fall-one.nc&data=02%7C01%7Calexandra.carter%40jcu.edu.au%7C69ed87af718b46f5bd9608d8637c2cf9%7C30a8c4e81ecd4f148099f73482a7adc0%7C0%7C0%7C637368732305044657&sdata=%2F2bzay2rkYGA5mhA70jI1DgTj1%2FQ0iHfPcldVQA9Qs4%3D&reserved=0) (Steven et al., 2019). Values for each data set are an aggregation of daily data from 12/2014 to 03/2019, which is then aggregated to monthly data and averaged over the year. Values for coastal sites outside the raster extent were estimated by IDW interpolation. These were all excluded from estuary analysis.
- **Wind speed** - *numeric data*. This was extracted from the eReefs 1 km grid hydrodynamic model representing wind speed (ms^-1^), available at: [https://data.aims.ereefs.org.au/thredds/fileServer/derived-download/gbr1_2.0/all-one/all-one.nc](https://aus01.safelinks.protection.outlook.com/?url=https%3A%2F%2Fdata.aims.ereefs.org.au%2Fthredds%2FfileServer%2Fderived-download%2Fgbr1_2.0%2Fall-one%2Fall-one.nc&data=02%7C01%7Calexandra.carter%40jcu.edu.au%7C69ed87af718b46f5bd9608d8637c2cf9%7C30a8c4e81ecd4f148099f73482a7adc0%7C0%7C0%7C637368732305044657&sdata=%2F2bzay2rkYGA5mhA70jI1DgTj1%2FQ0iHfPcldVQA9Qs4%3D&reserved=0). Wind speeds used by the eReefs models are derived from the Australian Bureau of Meteorology’s ACCESS data products (Bureau of Meteorology, 2020; Soldatenko et al., 2018; Steven et al., 2019) (Appendix S4; Fig. S1k). Values used here are an aggregation of daily data from 12/2014 to 03/2019, which is then aggregated to monthly data and averaged over the year. Values for coastal sites outside the raster extent were estimated by IDW interpolation. Wind speed was excluded from estuary analysis because of the effects of local topography that we could not account for.
- **Latitude** – *numeric data*. Environmental data useful to model estuarine seagrass distribution was limited, so latitude was included in the estuary models as a proxy for the north-south gradient in environmental conditions that was evident in the non-estuarine environmental data sets described above.

**Appendix S4.**

Figure S1. (a) Seagrass model boundaries (EI, estuary intertidal; ES, estuary subtidal; CI, coastal intertidal; CS, coastal subtidal; RI, reef intertidal; RS, reef subtidal). Red outline is the Great Barrier Reef World Heritage Area. (b–k) environmental variables used in random forest models to predict potential seagrass habitat, and multivariate regression trees to predict seagrass community types. Map created using ArcGIS software version 10.8 by Esri ([www.esri.com](http://www.esri.com)).


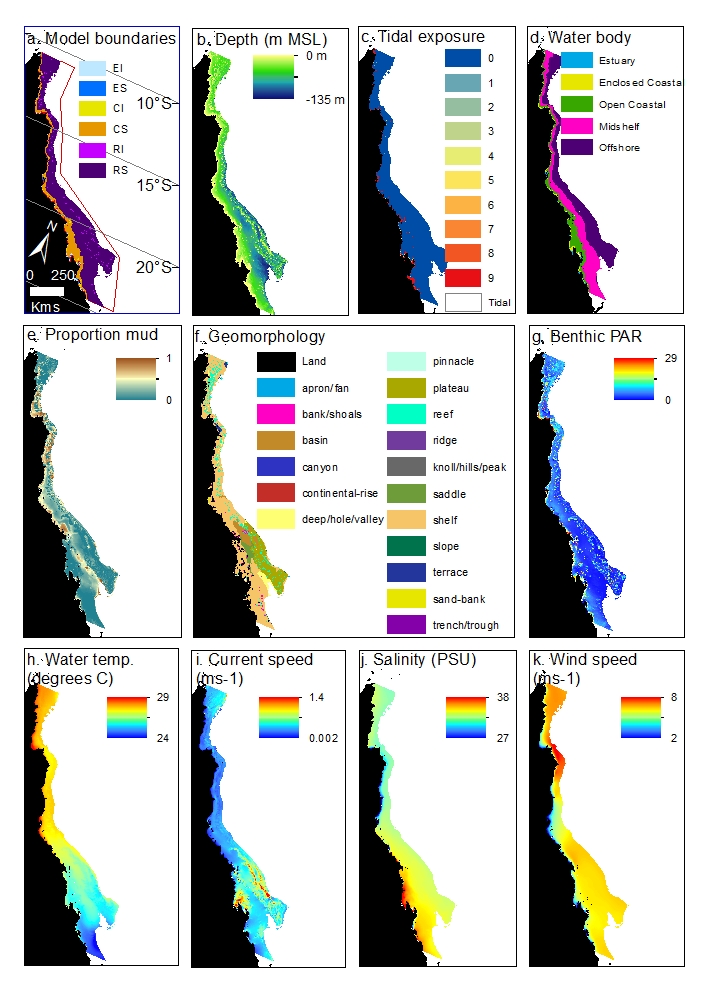


# References

Ayling, A. M., Roelofs, A. J., McKenzie, L. J., & Lee Long, W. J. (1997). Port of Cape Flattery Benthic Monitoring, Baseline Survey: Wet Season (February) 1996: Ports Corporation of Queensland, Brisbane.

Baird, M. E., Cherukuru, N., Jones, E., Margvelashvili, N., Mongin, M., Oubelkheir, K., Ralph, P. J., Rizwi, F., Robson, B. J., & Schroeder, T. (2016). Remote-sensing reflectance and true colour produced by a coupled hydrodynamic, optical, sediment, biogeochemical model of the Great Barrier Reef, Australia: comparison with satellite data. *Environmental modelling & software*, 78, 79-96.

Baird, M. E., Wild-Allen, K. A., Parslow, J., Mongin, M., Robson, B., Skerratt, J., Rizwi, F., Soja-Woźniak, M., Jones, E., & Herzfeld, M. (2020). CSIRO environmental modelling suite (EMS): scientific description of the optical and biogeochemical models (vB3p0). *Geoscientific Model Development*, 13(9), 4503-4553.

Beaman, R. J. (2017). *High-resolution depth model for the Great Barrier Reef - 30 m*. Retrieved from: <http://pid.geoscience.gov.au/dataset/115066>

Bishop-Taylor, R., Sagar, S., Lymburner, L., & Beaman, R. (2019). Between the tides: Modelling the elevation of Australia's exposed intertidal zone at continental scale. *Estuarine, Coastal & Shelf Science*, 223, 115-128.

Bryant, C., Wells, J., & Rasheed, M. (2019). Port of Townsville Annual Seagrass Monitoring Survey: October 2018. Centre for Tropical Water & Aquatic Ecosystem Research (TropWATER) Publication 19/01. James Cook University: Cairns.

Bureau of Meteorology. (2020). *Australian Community Climate and Earth-System Simulator (ACCESS) Numerical Weather Prediction (NWP) data*. Retrieved from: <http://www.bom.gov.au/nwp/doc/access/NWPData.shtml>

Campbell, S. J., Roder, C. A., McKenzie, L. J., & Lee Long, W. J. (2002). Seagrass Resources in the Whitsunday Region, 1999 and 2000: Northern Fisheries Centre, Cairns.

Carter, A., McKenna, S., Rasheed, M., Collier, C., McKenzie, L., Pitcher, R., & Coles, R. (2021). Synthesizing 35 years of seagrass spatial data from the Great Barrier Reef World Heritage Area, Queensland, Australia. *Limnology & Oceanography Letters*, 1-11. doi:10.1002/lol2.10193

Carter, A., & Rasheed, M. (2019). Mackay-Whitsunday Seagrass Monitoring 2018: Marine Inshore South Zone, Centre for Tropical Water & Aquatic Ecosystem Research Publication 19/05. James Cook University: Cairns.

Carter, A., Taylor, H., McKenna, S., & Rasheed, M. (2013). Critical Marine Habitats in High Risk Areas, Torres Strait – Seo Reef to Kai-Wareg Reef. James Cook University: Cairns.

Carter, A. B., Chartrand, K. M., & Rasheed, M. A. (2012). Critical marine habitats in high risk areas, Princess Charlotte Bay region - 2011 Atlas. Northern Fisheries Centre: Cairns.

Carter, A. B., McKenna, S. A., Rasheed, M. A., Collier, C., McKenzie, L., Pitcher, R., & Coles, R. (2020). *Seagrass mapping synthesis: A resource for coastal management in the Great Barrier Reef*. Retrieved from: <https://doi.org/10.25909/y1yk-9w85>

Carter, A. B., & Rasheed, M. A. (2014). Critical marine habitats in high risk areas, South Warden Reef to Howick Group - 2013 Atlas. James Cook University: Cairns.

Carter, A. B., & Rasheed, M. A. (2015). Critical marine habitats in high risk areas, Crescent Reef to Cape Flattery - 2014 Atlas. James Cook University: Cairns.

Chartrand, K., Wells, J., Carter, A., & Rasheed, M. (2019). Seagrasses in Port Curtis and Rodds Bay 2018: Annual long-term monitoring. James Cook University: Cairns.

Coles, R., McKenzie, L., De'ath, G., Roelofs, A., & Long, W. L. (2009). Spatial distribution of deepwater seagrass in the inter-reef lagoon of the Great Barrier Reef World Heritage Area. *MARINE ECOLOGY PROGRESS SERIES*, 392, 57-68. doi:<https://doi.org/10.3354/meps08197>

Coles, R. G. (1987). Seagrass Beds and Juvenile Prawn Nursery Grounds Between Bowen and Water Park Point: A Report to the Great Barrier Reef Marine Park Authority. Queensland Department of Primary Industries: Brisbane.

Coles, R. G., Lee Long, W. J., Helmke, S. A., Bennett, R. E., Miller, K. J., & Derbyshire, K. J. (1992). Seagrass beds and juvenile prawn and fish nursery grounds: Cairns to Bowen, Queensland. Northern Fisheries Centre: Cairns.

Coles, R. G., Lee Long, W. J., McKenzie, L. J., & Roder, C. A. (2002). Seagrass and the marine resources in the dugong protection areas of Upstart Bay, Newry region, Sand Bay, Ince Bay and the Clairview region. April/May 1999 and October 1999: Cairns.

Coles, R. G., Lee Long, W. J., Miller, K. J., Vidler, K. P., & Derbyshire, K. D. (1990). Seagrass beds and juvenile prawn and fish nursery grounds between Water Park Point and Hervey Bay, Queensland. Queensland Department of Primary Industries: Brisbane.

Coles, R. G., Lee Long, W. J., & Squire, L. C. (1985). Seagrass beds and prawn nursery grounds between Cape York and Cairns. Queensland Department of Primary Industries: Brisbane.

Coles, R. G., McKenzie, L. J., Rasheed, M. A., Mellors, J. E., Taylor, H., Dew, K., McKenna, S., Sankey, T. L., Carter, A. B., & Grech, A. (2007). Status and Trends of Seagrass Habitats in the Great Barrier Reef World Heritage Area. Reef and Rainforest Research Centre Limited: Cairns.

Dyall, A., Tobin, G., Creasey, J., Gallagher, J., Ryan, D. A., Heap, A. D., & Murray, E. (2004). *Queensland Coastal Waterways Geomorphic Habitat Mapping, Version 2 (1:100 000 scale digital data)*. Retrieved from: <http://catalogue.aodn.org.au/geonetwork/srv/eng/metadata.show?uuid=a05f7892-c344-7506-e044-00144fdd4fa6>

Geoscience Australia. (2017). *Intertidal Extents Model 25m. v. 2.0.0. Dataset*. Retrieved from: <https://ecat.ga.gov.au/geonetwork/srv/eng/catalog.search?node=srv#/metadata/7d6f3432-5f93-45ee-8d6c-14b26740048a>

Heap, A. D., & Harris, P. T. (2008). Geomorphology of the Australian margin and adjacent seafloor. *Australian Journal of Earth Sciences*, 55(4), 555-585.

Lee Long, W. J., McKenzie, L. J., & Coles, R. G. (1996a). Distribution of Seagrasses in Shoalwater Bay, Queensland - September 1995. Northern Fisheries Centre: Cairns.

Lee Long, W. J., Rasheed, M. A., McKenzie, L. J., & Coles, R. G. (1996b). Distribution of Seagrasses in Cairns Harbour and Trinity Inlet - December 1993. Northern Fisheries Centre: Cairns.

Lee Long, W. J., Roelofs, A. J., Coles, R. G., & McKenzie, L. J. (2001). Monitoring Oyster Point Seagrasses - 1995 to 1999. Report to the Great Barrier Reef Marine Park Authority. Northern Fisheries Centre: Cairns.

Margvelashvili, N., Andrewartha, J., Baird, M., Herzfeld, M., Jones, E., Mongin, M., Rizwi, F., Robson, B., Skerratt, J., & Wild-Allen, K. (2018). Simulated fate of catchment-derived sediment on the Great Barrier Reef shelf. *Marine Pollution Bulletin*, 135, 954-962.

McKenna, S., Rasheed, M., Reason, C., Wells, J., & Hoffman, L. (2019). Port of Abbot Point Long-Term Seagrass Monitoring Program - 2018. James Cook University: Cairns.

McKenzie, L. J., & Lee Long, W. J. (1996). Distribution and Abundance of Green Island Seagrass Meadows. CRC Reef: Townsville.

McKenzie, L. J., Lee Long, W. J., & Bradshaw, E. J. (1997). Distribution of Seagrasses in the Lizard Island Group - A Reconnaissance Survey, October 1995. Northern Fisheries Centre: Cairns.

McKenzie, L. J., Roder, C. A., & Yoshida, R. L. (2016). *Seagrass and associated benthic community data derived from field surveys at Low Isles, Great Barrier Reef, conducted July-August, 1997*. Retrieved from: <https://doi.org/10.1594/PANGAEA.858945>

McKenzie, L. J., Yoshida, R. L., & Unsworth, R. K. F. (2014). Disturbance influences the invasion of a seagrass into an existing meadow. *Marine Pollution Bulletin*, 86(1–2), 186-196. doi:<https://doi.org/10.1016/j.marpolbul.2014.07.019>

Pitcher, C. R., Doherty, P., Arnold, P., Hooper, J., Gribble, N., Bartlett, C., Browne, M., Campbell, N., Cannard, T., Cappo, M., Carini, G., Chalmers, S., Cheers, S., Chetwynd, D., Colefax, A., Coles, R., Cook, S., Davie, P., De'ath, G., Devereux, D., Done, B., Donovan, T., Ehrke, B., Ellis, N., Ericson, G., Fellegara, I., Forcey, K., Furey, M., Gledhill, D., Good, N., Gordon, S., Haywood, M., Hendriks, P., Jacobsen, I., Johnson, J., Jones, M., Kinninmoth, S., Kistle, S., Last, P., Leite, A., Marks, S., McLeod, I., Oczkowicz, S., Robinson, M., Rose, C., Seabright, D., Sheils, J., Sherlock, M., Skelton, P., Smith, D., Smith, G., Speare, P., Stowar, M., Strickland, C., Van der Geest, C., Venables, W., Walsh, C., Wassenberg, T., Welna, A., & Yearsley, G. (2007). Seabed Biodiversity on the Continental Shelf of the Great Barrier Reef World Heritage Area. AIMS/CSIRO/QM/QDPI CRC Reef Research Task Final Report.

Rasheed, M., & Roelofs, A. (1996). Distribution and abundance of Ellie Point seagrasses - December 1996. Unpublished report to the Trinity Inlet Management Program. Queensland Department of Primary Industries: Cairns.

Rasheed, M. A., Nguyen, T., Taylor, H. A., & Thomas, R. (2006). Critical Marine Habitats Adjacent to Hydrographers Passage, Great Barrier Reef, Queensland, Australia - 2006 Atlas. Northern Fisheries Centre: Cairns.

Rasheed, M. A., Reason, C. L., & Wells, J. N. (2019). Seagrass habitat of Cairns Harbour and Trinity Inlet: Annual Monitoring Report 2018. James Cook University: Cairns.

Rasheed, M. A., Roder, C. A., & Thomas, R. (2001). Port of Mackay seagrass, macro-algae and macro-invertebrate communities, February 2001. CRC Reef Research Centre: Townsville.

Rasheed, M. A., Thomas, R., Roelofs, A. J., & McKenna, S. A. (2005). Critical Marine Habitats Adjacent to the High Risk Inner Shipping Route in the Shelburne, Margaret and Indian Bays Region, Far North Queensland, Australia - 2005 Atlas. Northern Fisheries Centre: Cairns.

Roder, C. A., Lee Long, W. J., McKenzie, L. J., & Roelofs, A. J. (1998). Proposed Clump Point Boat Ramp and Facilities - Review of Marine Environment Factors (Seagrasses and Other Benthic Habitats). Unpublished Report to Queensland Department of Main Roads. Northern Fisheries Centre: Cairns.

Soldatenko, S., Tingwell, C., Steinle, P., & Kelly-Gerreyn, B. A. (2018). Assessing the impact of surface and upper-air observations on the forecast skill of the ACCESS numerical weather prediction model over Australia. *Atmosphere*, 9(1), 23.

Steven, A. D., Baird, M. E., Brinkman, R., Car, N. J., Cox, S. J., Herzfeld, M., Hodge, J., Jones, E., King, E., & Margvelashvili, N. (2019). eReefs: An operational information system for managing the Great Barrier Reef. *Journal of Operational Oceanography*, 12(sup2), S12-S28.

Taylor, H. A., McKenna, S. A., & Rasheed, M. A. (2010). Bustard Bay Seagrass Baseline Assessment: November 2009. DEEDI Publication, Fisheries Queensland: Cairns.

Wells, J., Reason, C., & Rasheed, M. (2019). Seagrass habitat of Mourilyan Harbour: Annual monitoring report - 2018. James Cook University: Cairns.

York, P., & Rasheed, M. (2019). Annual Seagrass Monitoring in the Mackay-Hay Point Region – 2018. James Cook University: Cairns.
